# Utilization of urea and chicken litter biochar to improve rice production

**DOI:** 10.1038/s41598-021-89332-y

**Published:** 2021-05-11

**Authors:** Nathaniel Maikol, Ahmed Osumanu Haruna, Ali Maru, Audrey Asap, Simon Medin

**Affiliations:** 1 Department of Crop Science, Faculty of Agricultural Science and Forestry, Bintulu Campus Universiti Putra Malaysia, 97008 Bintulu, Sarawak Malaysia; 2 Institut Ekosains Borneo, Sarawak Campus, Universiti Putra Malaysia Bintulu, 97008 Bintulu, Sarawak Malaysia; 3 Institute of Tropical Forestry and Forest Products (INTROP), Universiti Putra Malaysia, 43400 Serdang, Selangor Malaysia; 4 Institute of Tropical Agriculture and Food Security, Universiti Putra Malaysia, 43400 Serdang, Selangor Malaysia; 5 Institute of Agricultural Research, University of Ghana, P. O. Box 68, Accra, Ghana

**Keywords:** Plant sciences, Environmental sciences

## Abstract

The use of N fertilizers on tropical acid soils is increasing because of their low native fertility. Chicken litter biochar was used to improve N use efficiency and rice yield. The objective of this study was to determine the effects of chicken litter biochar on selected chemical properties of a tropical acid soil under rice (MR219) cultivation. Treatments evaluated were in this study were as follows: (1) T1, soil only, (2) T2, existing recommended fertilization, (3) T3, chicken litter biochar alone, and (4) T4, chicken litter biochar + existing recommended fertilization. Plant and soil analyses were conducted using standard procedures. The use of chicken litter biochar increased soil pH, total carbon, total P, available P, total N, and exchangeable N. Also, this practice decreased soil total acidity and exchangeable Al^3+^. Compared with T2, T4 significantly increased Crop Recovery Efficiency and Agronomic Recovery Efficiency of N. This resulted in a significant increase in the grain yield (11 t ha^−1^) of MR219 (Malaysia hybrid rice) for T4 compared with the existing rice grain yield of 5.9 t ha^−1^ (T2). Moreover, application of chicken litter biochar (5 t ha^−1^) to tropical acid soil suggested that N application can be reduced to 26.67%, 30.03%, 30.15%, and 14.15% of the recommended rates by MADA on days 10, 30, 50, and 70 after transplanting, respectively. Chicken litter biochar can improve the chemical properties of tropical acid soils and rice (MR219) grain yield.

## Introduction

Rice (*Oryza sativa* L.) is the most consumed grain crop in the world. The use of fertilizers in rice cultivation is higher in tropical soils because of their lower productivity^1,2^. Nitrogen plays an important role in rice production. From 2014 to 2017, the global N fertilizer demand increased from 3.7 to 8.8%, respectively. It is predicted that from 2018 to 2050, the world N consumption will be increasing at 9.5%^3,4^. The commonly used synthetic N fertilizer in agriculture is urea. It accounts for approximately 56% of the global N fertilizer consumption because it is cheaper and accessible^5^. However, ammonia volatilization from nitrogen fertilizers is serious in almost all agricultural systems^6,7^ particularly, in paddy fields^8^. Ammonia loss does not only reduce N use efficiency in rice fields, but it also increases the production cost of rice.

In this present study, it was assumed that increase in urea-N use efficiency will significantly mitigate N losses (ammonia volatilization and leaching). Approximately, 40% of all soluble N applied to soils are lost via ammonification, denitrification, and leaching^9^. In low land rice production on tropical mineral acid soils, ammonification is one of the major gas emission processes^10^. In the tropics, studies had been carried out to mitigate urea-N losses. For example, Ahmed et al.^11^ and Palanivell et al.^12^ used clinoptilolite zeolite to mitigate ammonia volatilization from aerobic and anaerobic soils. These authors also reported improvements in urea-N use efficiency and some soil chemical properties. However, accessibility to high quality clinoptilolite zeolite even for research purposes had remained a challenge. Another innovation is coating urea with humic acids to minimize urea-N loss through ammonia volatilization^11^ but this innovation is expensive for farmers to adopt.

The use of chicken litter biochar to mitigate N losses was recently explored^12^ and results demonstrated that this organic amendment does not only mitigate N losses but it also improves soil physico-chemical properties which in turn, increases N use efficiency. This is because chicken litter biochar reduces the detrimental effects of Al and Fe on plant roots. Also, this reaction enhances N uptake^13^. Additionally, biochars are rich in carboxylic and phenolic functional groups that have high affinity for ammonium ions to prevent these ions from being leached or volatilized^14,15^. Moreover, chicken litter biochar improves soil CEC, texture, cations, and anions^16^. The negatively charged surface of biochars enables them to adsorb ammonium ions. This chemical reaction prevents/protects absorbed ammonium ions from being converted to ammonia or leached from soils^12,17^. Therefore, the objectives of this study were to determine the effects of co-applying urea and chicken litter biochar on the:

1. Chemical properties of a tropical acid soil under rice (MR219) cultivation.
2. Nitrogen uptake, Crop Recovery Efficiency, Agronomic Recovery Efficiency of applied N, and grain yield of rice (MR219) plants.

## Materials and methods

### Brief information on the experimental area

**Figure 1:**
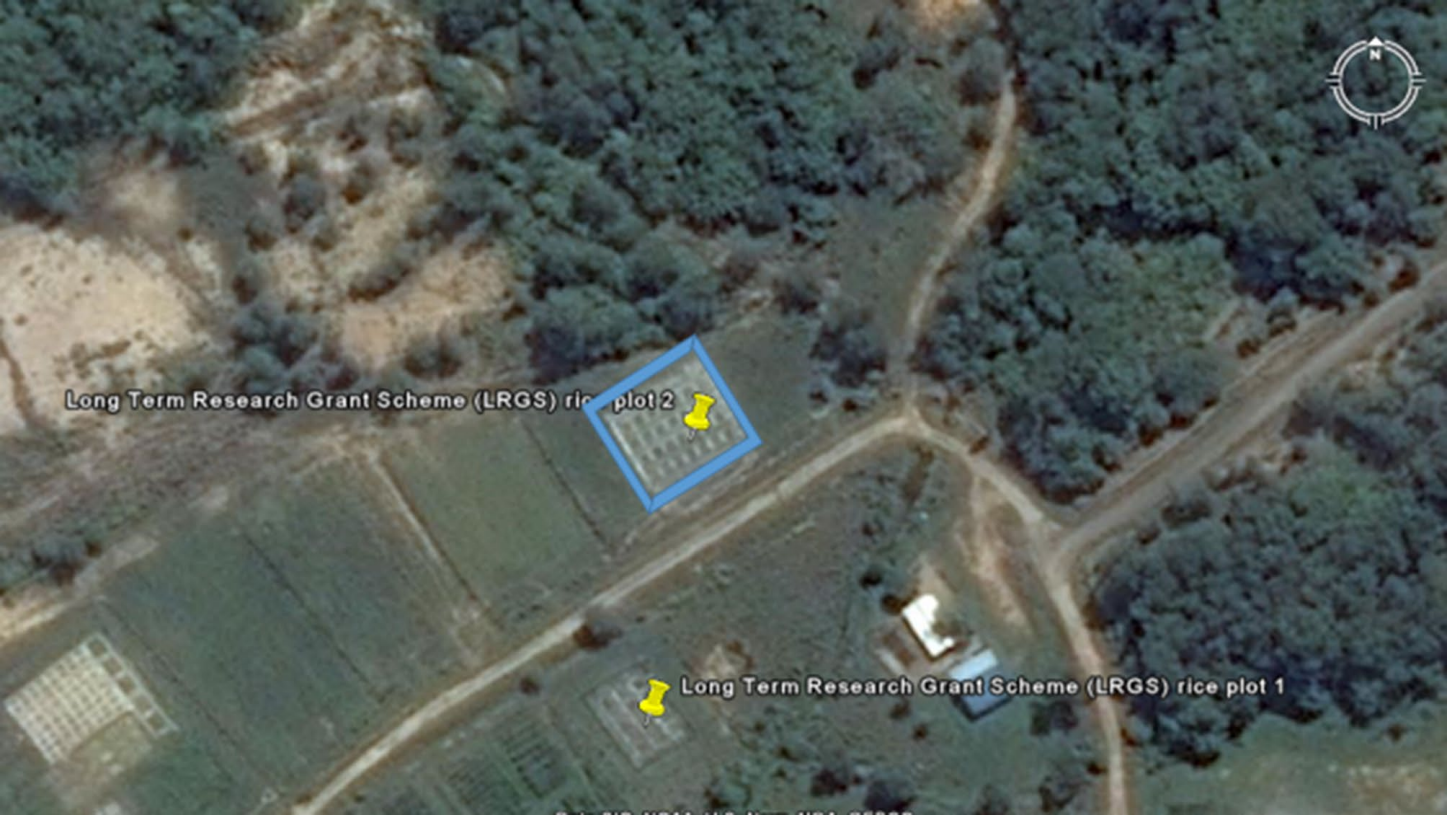
Aerial view of long-term research grant scheme rice plot two.

An experimental field called Long Term Research Grant Scheme (LRGS) Rice Plot Two on latitude 3° 12′ 54.48″ N and longitude 113° 05′ 39.03″ E was used (Fig. 1) for this present study. The area has an elevation of 102 ft. The soil of the experimental site is named Nyalau Series (*Typic Paleudults*) (Table 1). The experimental design which was used in this study was Randomized Complete Block Design with four blocks. Each plot size was 4 m^2^. The distance between the plots was 1 m and the distance between blocks was 2 m. The plots had PVC pipes to regulate the water levels in the plots. Rice plants in the plots were irrigated when necessary. The edges of the plots were covered with silver shine to control weeds and loss of soil (through high rainfall). The chicken litter biochar and urea rates used in this present study were based on 100 plant hill per plot (Table 2).

**Table 1:** Selected physical and chemical properties of *Typic Paleudults* (Nyalau Series).

Properties	Values	Properties	Values
pH in water	5.11 ± 0.02		cmol (+) kg^−1^
pH In KCl	3.97 ± 0.019		
	%	Available K	0.19 ± 0.003
Total carbon	2.55 ± 0.07	Total K	6.48 ± 0.71
Organic matter	4.40 ± 0.12		
Total N	0.103 ± 0.009	CEC	4.40 ± 0.06
	mg kg^−1^	Exchangeable Acidity	1.42 ± 0.009
Available NO_3_^−^	2.57 ± 0.23	Exchangeable Al^3+^	0.75 ± 0.014
Exchangeable NH_4_^+^	9.34 ± 0.47	Exchangeable H^+^	0.67 ± 0.006
Available P	3.00 ± 0.19	Exchangeable Cu^2+^	0.519 ± 0.009
Total P	58.29 ± 0.51	Exchangeable Mn^2+^	0.083 ± 0.0005
		Exchangeable Fe^2+^	48.31 ± 0.06
		Exchangeable Zn^2+^	2.25 ± 0.10
		Exchangeable Na^+^	1.40 ± 0.03
		Exchangeable Ca^2+^	1.55 ± 0.02
		Exchangeable Mg^2+^	0.0007 ± 0.000005
Texture (USDA)	Sandy loam

**Table 2:** Chicken litter biochar rates and fertilization schedule for field study.

Plant growth stages		Early tillering growth	Active growth	Formation of stalk	Grain filling
Days after transplanting		15–20	35–40	50–55	70–75
Treatments	g plot^−1^				
T1	0	0	0	0	0
T2	0	Mix A1	40 urea	Mix B1	Mix B1
T3	2000 biochar	0	0	0	0
T4	2000 biochar	Mix A1	40 urea	Mix B1	Mix B1

Mix A1 = (55 g Urea + 80 g ERP + 24 g MOP).

Mix B1 = (18 g Urea + 30 g ERP + 20 g MOP + 1.4 MgO).

ERP = Egyptian rock phosphate.

MOP = Muriate of potash.

The chicken litter biochar (BlackEarth Products) was imported from Australia. In this study, 5 tons biochar ha^−1^, the fertilizers, and their rates for the MR219 variety as recommended by Muda Agricultural Development Authority (MADA), Malaysia (MADA, 2015) were used (Table 3).

**Table 3:** Fertilization schedule recommended by Muda Agricultural Development Authority, 2015 for rice variety MR219.

Local rice variety MR219-105 to 111 days to maturity				
Plant growth stages	Early tillering growth	Active growth	Formation of stalk	Grain filling
Days after transplanting	15–20	35–40	50–55	70–75
Fertilizer type	Mixture fertilizers (Government Aid)	Urea (Government Aid)	Additional substance of fertilizer 12:12:17:2MgO + TE	Additional substance of fertilizer 12:12:17:2MgO + TE
Application rates (kg ha^−1^)	360 kg/ha	100 kg ha^−1^ (1 bag/alcove)	175 kg ha^−1^	175 kg ha^−1^

The mixture fertilizers (Government Aid) = 17.5 N: 15.5 P_2_O_5_: 10 K_2_O.

### Chemical properties of chicken litter biochar

The chicken litter biochar is produced by BlackEarth Products, Australia through pyrolysis (oxygen-limited conditions with high temperature). Chemical properties of the chicken litter biochar (Table 4) are consistent with Australia Certified Organic Standard, 2010. A 2000 g chicken litter biochar was applied to T3 and T4 through broadcasting after which it was thoroughly mixed with the soil using a shovel.

**Table 4:** Selected physical and chemical properties of chicken litter biochar.

Physical and chemical properties					
pH	8.5	Av. particle size	0.5–2 mm		
Ash content (%)	23.7				
Macro nutrients		Micro nutrients			
Total Carbon (%)	63.7	Mg kg^−1^			
Fixed Carbon (%)	61.2	Silicon	2.3	Magnesium oxide	6.7
Nitrogen (%)	2.8	Aluminium	1.5	Arsenic	2.1
Phosphate (%)	2.6	Potassium oxide	16.3	Cadmium	0.7
Potassium (%)	3.9	Boron	62	Chromium	9.6
Calcium (%)	5.9	Copper	167	Mercury	0.06
Sulphur (%)	0.59	Manganese	1130	Nickel	14
		Zinc	856	Lead	12

*Source*: BlackEarth Company in north of Bendigo Victoria, Australia.

A 2000 g chicken litter biochar was applied to the plots that were labelled T3 (Chicken litter biochar only) and T4 (Chicken litter biochar with existing fertilization) by broadcasting the chicken litter biochar (T4) on the surface of the soil. Thereafter, the chicken litter biochar and the soil were mixed thoroughly and levelled using a shovel.

### Watering and transplanting

A day before transplanting the MR219 seedlings, 16 plots were watered to 1.5 cm. A 15 day old nursed rice seeds (MR219 variety) were transported to the field a day before to enable them to adapt to the field condition. Thereafter, 100 hills (3 seedlings per hill) of the MR219 seedlings were transplanted in the experimental plots. Afterwards, water in the plots was maintained at 1.5 cm above the soil surface until the rice seedlings were established (14 days after transplanting) after which the water level was increased to approximately 2.5–4 cm. This water level was maintained till the end of the field study.

### Fertilization, weeding, and pest control

The chemical fertilizers as recommended by MADA (Table 3) were broadcast in the plots on 15, 35, 50, and 70 days after transplanting. Weeding of the plots was carried out manually (hand weeding) whereas grasshoppers, stem borers, and caterpillars were sprayed with Halex Malathion 84 EC.

### Harvesting of rice plants

Rice plants were harvested at maturity (99–111 days after transplanting) due to the positive effects of the treatments with the chicken litter biochar on early grain ripening (enhanced vigorous growth and development). Sampling was done every 10 days after transplanting for 111 days (MR219 life span). Three rice plant hills were selected at random and this excluded border plants. Plant height was measured from the soil surface to the tip of the tallest leaf using a measuring tape. Number of tillers was counted for each hill and number of leaves was counted by counting the leaves of each tiller in a hill. Culm height was measured from the soil surface to the culm of the panicle of the tallest tiller using a measuring tape. Number of panicles for each of the 10 rice plant hills (randomly selected) were counted after which 10 panicles were randomly harvested into separate plastic bags for total grain counting, grain filling, and 1000 grain weight determination. Afterwards, the three rice plant hills were harvested for dry matter yield (every 10 days until maturity of the MR219 variety paddy for 111 days). Thereafter, the rest of the rice plants were harvested on the next day and panicles were cut using a pair of scissors. The harvested panicles were transferred to the laboratory, air dried at room temperature, after which the rice grains were removed.

### Soil sampling and analysis

Soil samples were taken at five points using diagonal method (because of the plot size, 4 m^2^). The soil samples were taken every 10 days (using auger) for 111 days after which they were prepared and analyzed. Soil pH was determined in a 1:2.5 (soil: distilled water) using a digital pH meter^18^. Soil total C was calculated as 58% of the organic matter determine using loss of weight on ignition^19^. The cation exchange capacity (CEC) was determined using leaching method^20^ followed by steam distillation^21^. Exchangeable cations were extracted with 1 M NH_4_OAc (pH 7) using the leaching method^20^. Thereafter, the extracted samples were determined using Atomic Absorption Spectrometery (AAnalyst 800, PERKIN Elmer Instruments, Norwalk, CT). Total N was determined using Kjeldhal method^22^ and inorganic N (NO_3_^-^ and NH_4_^+^) using Keeney and Nelson^23^. Soil total P and K were extracted using aqua regia method after which total P was determined using a Spectrophotometer after blue colour was developed using the Blue Method^24^ whereas total K was determined using Atomic Absorption Spectrometry (AAnalyst 800, Perkin Elmer Instrument, Norwalk, CT). Soil exchangeable acidity, H^+^, and Al^3+^ were determined using acid–base titration method^25^.

### Aboveground biomass analysis

Aboveground biomass samples were digested using the Single Dry Ashing Method^20^ and K, Ca, Mg, Mn, Zn, Fe, and Cu were determined using Atomic Absorption Spectrometry (AAS) whereas P was determined using Blue method^24^. Total N was determined using Kjeldhal method^26^. The nutrient concentrations were multiplied by the dry matter yield of the rice plants to quantify nutrient uptake. The agronomic and crop recovery efficiency of the applied urea were determined using the listed equations.

### Agronomic and crop recovery efficiency of applied nitrogen

The Agronomic and Crop recovery efficiency of applied urea-N^27^ was determined using the formulae as follows:

Agronomic recovery efficiency

- AE_N_ = (Y_N_ − Y_0_)/F_N_ respectively where
- F_N_—amount of (fertilizer) N applied (kg ha^−1^)
- Y_N_—crop yield with applied N (kg ha^−1^)
- Y_0_—crop yield (kg ha^−1^) in a control treatment with no N,

Crop recovery efficiency

- RE_N_ = (U_N_ − U_0_)/F_N_ respectively where
- F_N_—amount of (fertilizer) N applied (kg ha^−1^)
- U_N_—total plant N uptake in aboveground biomass at maturity (kg ha^−1^) in a plot that received N,
- U_0_—the total N uptake in aboveground biomass at maturity (kg ha^−1^) in a plot that received no N.

### Determination of grain yield

The total rice grain yield was determined using the method described by Matsushirna and Tanaka^28^

1 $${\text{Yield}} = \frac{{{\text{weight}}\;{\text{of}}\;1000\;{\text{grain}}\; \times \;{\text{spikelet}} \times \% \;{\text{total}}\;{\text{grain}}\;{\text{filled}}}}{{10000\,{\text{m}}^{2} \times 1000}}$$

where the area for 1 hectare = 10,000 m^2^ was used to enable the yield to be expressed in hectare and the 1000 was used to get the dry weight of 1 grain.

### Weight of one thousand rice grains determination

A 1000 matured rice grains (well filled) from 10 panicles which were harvested in each plot were placed in a clean crucible. The crucibles with the grains were oven dried at 60 °C until constant weight was attained after which the samples were cooled in a desiccator. The dried grains were gently transferred into a beaker and weighed.

2 $${\text{Dry}}\;{\text{weight}}\;1\;{\text{grain}} = \frac{{{\text{dry}}\;{\text{weight}}\;{\text{of}}\;1000\;{\text{grains}}}}{ 1000 }$$

### Spikelet quantification

3 $${\text{Spikelet}} = \frac{{{\text{number}}\;{\text{of}}\;{\text{panicles}}\;{\text{per}}\;{\text{hill}} \times \% \;{\text{total}}\;{\text{grain}}\;{\text{filling}}}}{{{\text{area}}\;{\text{per}}\;{\text{hill}} }}$$

The number of panicles hill^−1^ was determined on the field after number of panicles for the 10 randomly selected hills were counted and the average of the 10 for each plot was taken. Percentage grain filling was determined by checking filled and uilled grains for each of the 10 randomly harvested panicles.

4 $$\% \;{\text{total}}\;{\text{grain}}\;{\text{filling}} = \frac{{{\text{Total}}\;{\text{number}}\;{\text{of}}\;{\text{grain}}\;{\text{filled}}}}{{{\text{Total}}\;{\text{grain}}\;{\text{of}}\;{\text{the}}\;{\text{panicle}}}} \times 100$$

### Statistical analysis

Analysis of variance (ANOVA) was used to test treatment effects whereas treatments means were compared using Tukey’s Test. Statistical Analysis Software version 9.3 was used for the statistical analysis^29^.

## Result and discussion

### Effect of chicken litter biochar on soil carbon

Decrease in total carbon indicates decline in soil organic matter and the associated deterioration of one or more soil functions^30^. Decreasing soil total carbon particularly in tropical acid soils rapidly reduces soil productivity especially in terms of nutrient retention. Application of soil organic amendments such as chicken litter biochar improves soil organic matter and carbon content^31^. In this present study, T3 and T4 significantly increased soil total carbon compared with the plots without (T1 and T2) the chicken litter biochar (Table 5). Apart from the chicken litter biochar creating a conducive environment for microbes to thrive, it also made the soil relatively less compact for the rice plants’ root biomass development. The soil total carbon (especially T3 and T4) decreased with increasing days after transplanting because of the decomposition of the chicken litter biochar by the microbes in the soil^32^. For the soil without the chicken litter biochar (T1 and T2), total carbon decreased because of the decomposition of soil organic matter unlike the recalcitrant chicken litter biochar. Moreover, because the present study was conducted during the wet it was possible that some of the existing organic matter in the plots without the chicken litter biochar got leached out of the soil profile.

**Table 5:** Chicken litter biochar on total carbon in one hundred and eleven days growth of MR219.

Days of sampling	Treatments			
	T1	T2	T3	T4
	%			
Initial	2.15^b^ ± 0.08	2.06^b^ ± 0.06	2.49^a^ ± 0.10	2.44^a^ ± 0.05
10	2.15^c^ ± 0.08	2.23^c^ ± 0.06	2.58^a^ ± 0.07	2.38^b^ ± 0.07
20	1.83^b^ ± 0.06	1.86^b^ ± 0.05	2.29^a^ ± 0.06	2.32^a^ ± 0.07
30	1.86^c^ ± 0.05	1.77^c^ ± 0.06	2.41^a^ ± 0.06	2.03^b^ ± 0.03
40	2.00^bc^ ± 0.14	1.83^c^ ± 0.06	2.26^ab^ ± 0.03	2.38^a^ ± 0.07
50	1.77^b^ ± 0.11	1.71^b^ ± 0.09	2.23^a^ ± 0.16	2.18^a^ ± 0.15
60	1.86^b^ ± 0.05	2.00^b^ ± 0.06	2.29^a^ ± 0.09	2.23^a^ ± 0.06
70	1.80^ab^ ± 0.03	1.42^c^ ± 0.03	1.68^b^ ± 0.03	1.83^a^ ± 0.06
80	1.68^c^ ± 0.10	1.57^c^ ± 0.03	1.94^b^ ± 0.09	2.20^a^ ± 0.08
90	1.83^b^ ± 0.06	1.68^b^ ± 0.07	2.35^b^ ± 0.09	2.15^a^ ± 0.15
100	1.51^b^ ± 0.05	1.65^a^ ± 0.06	–	–
111	1.68 ± 0.07	–	–	–

Different letters within a row indicates significant difference between means of four replicates ± standard error using Tukey’s test at *p* ≤ 0.05.

Chicken litter biochar is a stable and carbon-rich material which when added to soils, it increases soil total carbon^33^. Soil total carbon which is one of the most important soil properties affects soil productivity because carbon as soil organic matter alters the physical, chemical, and biological properties of most soils. This is one of the reasons why soil carbon is used to indicate soil quality^34^. Improved soil organic carbon improves aggregation of soil particles resulting in improved soil structure, soil bulk density, and root growth^35^. Higher soil organic carbon enables movement of air and plants nutrients to plant roots. In addition, higher soil organic matter decreases soil crusting besides improving water infiltration rate to enhance plant productivity^36,37^. Chemically, soil total carbon increases cation exchange capacity (CEC) of soils and 20–80 percent of the CEC of soils is related to soil organic matter^38,39^. These cation exchange sites are important for retention of nutrients because the soil organic carbon binds N and P but these N and P are released upon decomposition. Moreover, soil organic carbon enhances chelation of metals including trace elements. This reaction increases bioavailability of trace elements required for plant growth^40,41^. Additionally, soil organic carbon provides binding sites for Al and Fe ions in acidic soils. This reaction reduces the toxic effects Al and Fe ions on plant roots.

### Chicken litter biochar on pH and exchangeable acidity of a tropical acid soil

Ultisols are made up of feldspars and micas that are highly weathered. During high rainfall, their base cations such as Ca^2+^, Mg^2+^, Na^+^, and K^+^ are leached^10,42^. With time, these base cations are replaced with Al and Fe ions whose hydrolysis increases the acidity of Ultisols. Subsoil acidity as a result of high Al:Ca ratio makes it difficult for most agronomic crops to extend their roots to subsoil^43^. Ch’ng et al.^44^ and Maru et al.^45^ found that amending a tropical acid soil with chicken litter biochar increased soil pH, exchangeable acidity, exchangeable Al, and H^+^. In this present study, the effects of chicken litter biochar on pH, exchangeable Al, H^+^, and acidity were monitored at ten days interval for 110 days.

Before treatments were applied, the soil pH in soluble water and KCl for the plots that were assigned T1, T2, T3, and T4 were similar. However, 10 days after applying the chicken litter biochar, pH of the plots with T4 were similar to those with T3 but significantly higher than those of T1 and T2 (Tables 6, 7). This may be that the chicken litter biochar was able to fix Al and Fe to prevent them from being hydrolysed to produce H^+^ ions, suggesting that amending a tropical acid soil with chicken litter biochar can minimize Al and Fe hydrolysis^46^. When urea was applied to the plots of T2 at the fifteenth day after transplanting, their pH were similar to those of T3 and T4 (Tables 6, 7). There was an improvement in soil pH following the application of urea (T2) because urea hydrolyzes to release OH^-^ ions^47^. Although the chicken litter biochar and urea increased the soil pH, the pH of T3 and T4 decreased with increasing time because the chicken litter biochar decomposed with increasing time. Before the treatments were applied, the results revealed that exchangeable acidity of T2 was significantly higher compared those of T1, T3, and T4 but those of T1 and T3 were significantly higher than that of T4. However, on the 10th day after transplanting, the exchangeable acidity of T4 was similar to that of T3 but significantly lower than those of T1 and T2 (Table 8). Also, before the treatments were applied, the soil exchangeable Al^3+^ of T2 was significantly higher than those of T1, T3, and T4 (Table 9) but on the 10th day after transplanting, the exchangeable Al^3+^ of T1 was significantly higher than those of T2, T3, and T4. On 20 day after transplanting, the exchangeable Al^3+^ of T3 and T4 were not detected because the chicken litter biochar was able fix the exchangeable Al^3+^. Before transplanting, the soil exchangeable H^+^ of T1, T2, T3, and T4 were similar. However, 10 days after transplanting, T1 and T2 showed significantly higher H^+^ ions than those of T3 and T4 (Table 10). On day 20 after transplanting, T1 demonstrated higher exchangeable H^+^ than those of T2, T3, and T4. From day 40 to day 80 after transplanting, the exchangeable H^+^ among all the treatments were similar. However, after 90 days, the H^+^ ions of T1, T2, and T4 were significantly higher than that of T3. On days 100 and 110 after transplanting, the H^+^ ions of T1 and T2 were similar.

**Table 6:** Chicken litter biochar on soil pH in KCl at one hundred and eleven days after transplanting of MR219.

Days of sampling	Treatments			
	T1	T2	T3	T4
Initial	4.16^a^ ± 0.08	4.29^a^ ± 0.11	4.36^a^ ± 0.13	4.41^a^ ± 0.10
10	4.11^b^ ± 0.09	4.29^b^ ± 0.10	4.47^ab^ ± 0.12	4.73^a^ ± 0.14
20	3.84^b^ ± 0.06	3.96^ab^ ± 0.06	3.99^ab^ ± 0.06	4.12^a^ ± 0.03
30	3.88^b^ ± 0.06	3.98^ab^ ± 0.05	3.98^ab^ ± 0.07	4.12^a^ ± 0.01
40	3.87^b^ ± 0.07	3.96^ab^ ± 0.06	3.94^ab^ ± 0.09	4.12^a^ ± 0.02
50	3.84^b^ ± 0.06	3.96^ab^ ± 0.06	3.97^ab^ ± 0.08	4.10^a^ ± 0.02
60	3.87^b^ ± 0.06	3.97^ab^ ± 0.06	3.98^ab^ ± 0.08	4.10^a^ ± 0.03
70	3.85^a^ ± 0.07	3.90^a^ ± 0.09	3.95^a^ ± 0.11	4.10^a^ ± 0.03
80	3.85^b^ ± 0.08	3.95^ab^ ± 0.05	3.92^ab^ ± 0.09	4.09^a^ ± 0.03
90	3.73^b^ ± 0.04	3.88^ab^ ± 0.07	3.88^ab^ ± 0.09	4.03^a^ ± 0.009
100	3.79^a^ ± 0.06	3.88^a^ ± 0.08	–	–
111	3.77 ± 0.09	–	–	–

Different letters within a row indicates significant difference between means of four replicates ± standard error using Tukey’s test at *p* ≤ 0.05.

**Table 7:** Chicken litter biochar on soil pH in water at one hundred and eleven days after transplanting of MR219.

Days of sampling	Treatment			
	T1	T2	T3	T4
Initial	4.99^a^ ± 0.21	5.28^a^ ± 0.37	5.49^a^ ± 0.16	5.82^a^ ± 0.09
10	4.93^b^ ± 0.23	5.38^ab^ ± 0.24	5.40^ab^ ± 0.33	5.93^a^ ± 0.13
20	4.26^b^ ± 0.11	4.58^ab^ ± 0.28	4.48^ab^ ± 0.21	4.95^a^ ± 0.16
30	4.46^b^ ± 0.18	4.69^ab^ ± 0.15	4.63^ab^ ± 0.18	5.09^a^ ± 0.06
40	4.59^a^ ± 0.18	4.77^a^ ± 0.24	4.81^a^ ± 0.27	5.20^a^ ± 0.15
50	4.55^a^ ± 0.19	4.80^a^ ± 0.27	4.77^a^ ± 0.25	4.98^a^ ± 0.09
60	4.55^a^ ± 0.19	4.71^a^ ± 0.23	4.75^a^ ± 0.22	4.97^a^ ± 0.12
70	4.53^a^ ± 0.22	4.55^a^ ± 0.24	4.57^a^ ± 0.29	4.76^a^ ± 0.21
80	4.58^a^ ± 0.16	4.74^a^ ± 0.15	4.59^a^ ± 0.20	5.00^a^ ± 0.07
90	4.33^a^ ± 0.09	4.75^a^ ± 0.19	4.63^a^ ± 0.20	4.87^a^ ± 0.05
100	4.42^a^ ± 0.14	4.70^a^ ± 0.27	–	–
111	4.38 ± 0.14	–	–	–

Different letters within a row indicates significant difference between means of four replicates ± standard error using Tukey’s test at *p* ≤ 0.05.

**Table 8:** Chicken litter biochar on exchangeable acidity at one hundred and eleven days after transplanting of MR219.

Days of sampling	Treatments			
	T1	T2	T3	T4
	cmol kg^−1^			
Initial	0.75^b^ ± 0.018	0.86^a^ ± 0.02	0.74^b^ ± 0.02	0.45^c^ ± 0.02
10	1.03^a^ ± 0.06	0.92^b^ ± 0.02	0.47^c^ ± 0.02	0.43^c^ ± 0.01
20	1.91^a^ ± 0.05	1.49^b^ ± 0.04	0.89^c^ ± 0.009	0.91^c^ ± 0.01
30	1.51^a^ ± 0.06	1.38^a^ ± 0.08	1.31^a^ ± 0.09	0.84^b^ ± 0.02
40	1.50^a^ ± 0.06	1.29^b^ ± 0.06	0.82^c^ ± 0.05	0.75^c^ ± 0.03
50	1.46^a^ ± 0.03	1.28^b^ ± 0.09	0.80^c^ ± 0.03	0.81^c^ ± 0.01
60	1.40^a^ ± 0.04	1.37^a^ ± 0.09	0.86^b^ ± 0.06	0.81^b^ ± 0.03
70	1.13^a^ ± 0.04	1.24^a^ ± 0.01	0.71^b^ ± 0.06	0.72^b^ ± 0.04
80	1.25^a^ ± 0.05	1.15^a^ ± 0.13	1.04^a^ ± 0.09	0.70^b^ ± 0.07
90	1.51^a^ ± 0.05	1.23^b^ ± 0.05	0.85^c^ ± 0.09	0.81^c^ ± 0.03
100	1.12^a^ ± 0.12	1.13^a^ ± 0.07	–	–
111	1.09 ± 0.03		–	–

Different letters within a row indicates significant difference between means of four replicates ± standard error using Tukey’s test at *p* ≤ 0.05.

**Table 9:** Chicken litter biochar on exchangeable Al^3+^ at one hundred and eleven days after transplanting of MR219.

Days of sampling	Treatments			
	T1	T2	T3	T4
	cmol kg^−1^			
Initial	0.32^b^ ± 0.02	0.48^a^ ± 0.04	0.30^b^ ± 0.01	0.18^c^ ± 0.02
10	0.52^a^ ± 0.03	0.45^b^ ± 0.02	0.24^c^ ± 0.02	0.15^d^ ± 0.02
20	0.50^b^ ± 0.03	0.79^a^ ± 0.05	ND	ND
30	0.50^a^ ± 0.01	0.40^b^ ± 0.01	ND	ND
40	0.62^a^ ± 0.04	0.42^b^ ± 0.02	ND	ND
50	0.52^a^ ± 0.04	0.46^a^ ± 0.02	ND	ND
60	0.43^a^ ± 0.05	0.54^a^ ± 0.04	ND	ND
70	0.33^a^ ± 0.03	0.33^a^ ± 0.13	ND	ND
80	0.46^a^ ± 0.04	0.38^b^ ± 0.03	ND	ND
90	0.49^a^ ± 0.08	0.37^a^ ± 0.05	ND	ND
100	0.30^a^ ± 0.08	0.42^a^ ± 0.05	–	–
111	0.27 ± 0.03	–	–	–

Different letters within a row indicates significant difference between means of four replicates ± standard error using Tukey’s test at *p* ≤ 0.05. ND = Not detected.

**Table 10:** Chicken litter biochar on exchangeable H^+^ at one hundred and eleven days after transplanting of MR219.

Days of sampling	Treatments			
	T1	T2	T3	T4
	cmol kg^−1^			
Initial	0.43^a^ ± 0.01	0.38^a^ ± 0.06	0.44^a^ ± 0.02	0.27^a^ ± 0.01
10	0.51^a^ ± 0.03	0.47^a^ ± 0.01	0.23^b^ ± 0.02	0.28^b^ ± 0.02
20	1.41^a^ ± 0.06	0.70^b^ ± 0.01	0.78^b^ ± 0.11	0.88^b^ ± 0.05
30	1.01^b^ ± 0.06	1.00^b^ ± 0.08	1.19^a^ ± 0.05	0.76^c^ ± 0.07
40	0.88^a^ ± 0.08	0.87^a^ ± 0.04	0.65^a^ ± 0.18	0.75^a^ ± 0.03
50	0.94^a^ ± 0.04	0.82^a^ ± 0.09	0.69^a^ ± 0.12	0.81^a^ ± 0.01
60	0.97^a^ ± 0.08	0.83^a^ ± 0.12	0.70^a^ ± 0.18	0.81^a^ ± 0.03
70	0.80^a^ ± 0.05	0.90^a^ ± 0.13	0.71^a^ ± 0.06	0.72^a^ ± 0.04
80	0.79^a^ ± 0.04	0.77^a^ ± 0.10	0.90^a^ ± 0.03	0.70^a^ ± 0.07
90	1.03^a^ ± 0.07	0.87^ab^ ± 0.01	0.64^b^ ± 0.16	0.81^ab^ ± 0.03
100	0.82^a^ ± 0.05	0.71^a^ ± 0.06	–	–
111	0.82 ± 0.01	–	–	–

Different letters within a row indicates significant difference between means of four replicates ± standard error using Tukey’s test at *p* ≤ 0.05.

Generally, the exchangeable acidity of T3 and T4 were lower compared those of T1 and T2 because the chicken litter biochar improved the soil functional groups to fix Al^3+^ and Fe^3+^. Functional groups such as COOH, OH, and ketone are able to fix toxic substances such Al, Fe, and Mn in soils because of their high affinity for these ions^48^. From days 20–90 after transplanting, the exchangeable acidity for all of the treatments decreased. The exchangeable Al^3+^ especially those of T1 and T2 decreased with increasing days after transplanting. This explains why the soil exchangeable acidity decreased with increasing days after transplanting. The chicken litter biochar was able to increase the soil pH due to its alkalinity and high pH buffering capacity. Yuan and Xu^2^ found that increase in soil pH strongly correlated with biochar alkalinity (R^2^ = 0.95) than without biochar pH (R^2^ = 0.46). Additionally, cations (Ca, K, Mg, Na and Si) of the chicken litter biochar commonly form carbonates and oxides during pyrolysis^49,50^. These carbonates and oxides react with the H^+^ and monomeric Al species in acid soils. This reaction does not only increase soil pH but it also decreases soil exchangeable acidity^51^.

Furthermore, Yuan et al.^15^ demonstrated that the functional groups of the chicken litter biochar (COO^−^ and O^−^) react with soil H^+^. The ability of the chicken litter biochar to buffer soil pH ameliorates soil acidity because of the resulting increase in protonation-deprotonation of the functional groups of the chicken litter biochar^52^. Aluminum toxicity limits plant root growth and crop productivity in acid soils. Free Al^3+^ at high concentration inhibits root cell expansion, elongation, and division^53–55^, resulting in small root systems to impede water and nutrient uptake. Biochars are capable of decreasing Al bioavailability in acid soils. They can also alleviate Al toxicity to plants. Alling et al.^56^ reported that after applying biochar, the concentration of Al^3+^ in soil leachate decreased from approximately 2 mg L^−1^ to undetectable levels. Furthermore, the carboxylic functional groups of the biochar provided additional binding sites for Al^3+^ besides the inorganic components and functional oxygen groups of the chicken litter biochar^6^. Moreover, the higher surface area and pores of biochars provide more adsorption sites for sorption of Al and other metals^57^.

### Influence of chicken litter biochar on total and exchangeable nitrogen and phosphorus of a tropical acid soil under low land rice cultivation

**Figure 2:**
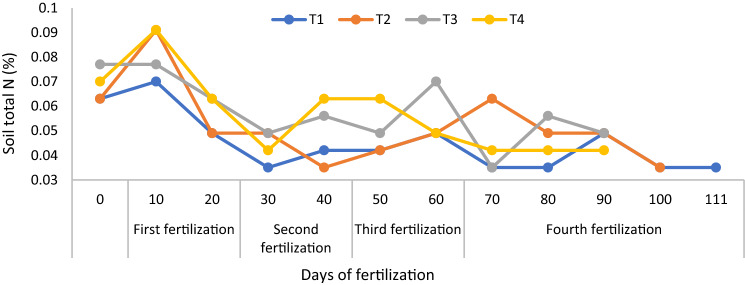
Improving soil total nitrogen concentration using chicken litter biochar in MR219 rice cultivation.

Amending tropical acid soils with chicken litter biochar directly contributes to plant nutrient availability because of the charge interactions on the surface of this biochar. Also, amending acid soils with chicken litter biochar decreases soil acidity and Al toxicity to unlock fixed P^58^. The soil total N for T3 and T4 were higher than with T1 and T2 (Fig. 2). On the 10th day after transplanting, the soil total N of T2 and T4 were similar but higher than those of T1 and T3. On the 15th days after transplanting, first fertilization applied to T2 and T4, the total N in the soil was more in the soil T4 compared to T2 (Fig. 2) because T4 was amended with chicken litter biochar. This trend continued until Day 40 after 35 days from the first fertilization application with T4 causing significant amount of total N in the soil (Fig. 2). The T4 rice plant reached maturity earlier because of the higher absorption of N by the rice plants in T4 (Fig. 2).

**Figure 3:**
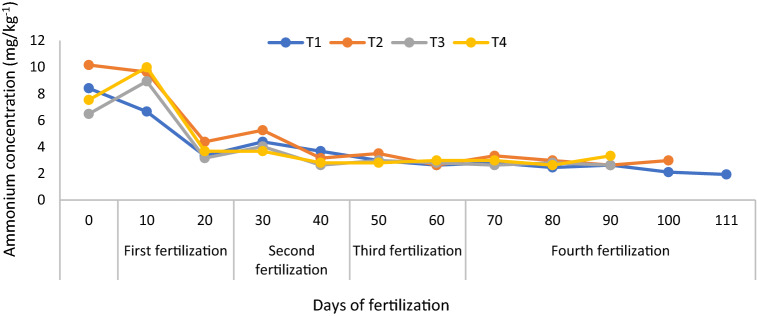
Improving soil ammonium content availability using chicken litter biochar in MR219 rice cultivation.

**Figure 4:**
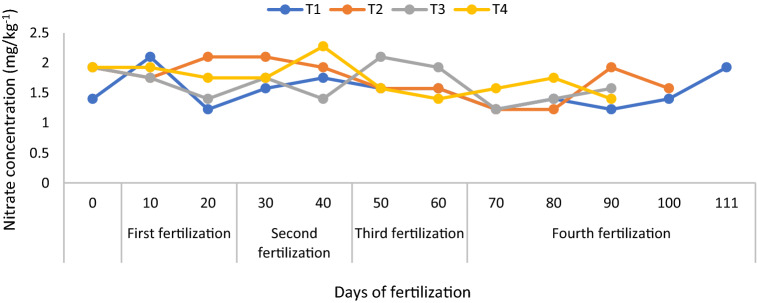
Improving soil nitrate content using chicken litter biochar in MR219 rice cultivation.

At 20 days after transplanting, T2 and T4 did not significantly increase soil total N (Fig. 2) because of the higher absorption of N by the rice plants. Soil exchangeable NH_4_^+^ (regardless of treatments) from 20 days after transplanting to 110 days were also similar and lower than those for the first 10 days after transplanting, because of poorer root development and active N absorption by the rice plants (Fig. 3). At 10 days after transplanting, a rapid decline in the soil exchangeable NH_4_^+^ was also observed due to stalk formation stage of the rice plants. This finding is consistent with that of the soil total N. The soil exchangeable NO_3_^-^ of T1 was lower than those of T2, T3, and T4 but those of T2 and T4 were higher all throughout the growth and development phase of the rice plants (Fig. 4).

The chicken litter biochar improved soil total P availability and the results of this study demonstrate that the soil total P of T3 was significantly higher than those of T1, T2, and T4 (Table 11). Between 10 and 30 days after transplanting, the total P of T4 was higher compared with those of T1, T2, and T3. Biochar application in tropical soils considerably improves soil fertility by increasing soil pH as well as reducing Al and Fe. This reaction unlocks fixed P for plant use^59^. Between 40 and 50 days after transplanting, the soil total P of T3 was higher than those of T1, T2, and T4. At 60 days after transplanting, the soil total P of T3 and T4 were similar but significantly higher than those of T1 and T2. At days 70 and 80, the soil total P of T4 was significantly higher than those of T1, T2, and T3 whereas at day 90, total P of T3 was significantly higher than those of T1, T2, and T4. Generally, the total P of T3 and T4 decreased with increasing days after transplanting because of improved plant root development and P absorption by the rice plants. The available P of T2 and T4 were similar but significantly higher compared with those of T1 and T3 (Table 12). From days 10–50 after transplanting, T4 was higher compared with T1, T2, and T3. At 60 day, T3 was significantly higher than those of T1, T2, and T4, but from days 70–90, the available P of T4 was significantly higher than those of T1, T2, and T3.

**Table 11:** Chicken litter biochar on total phosphorus at one hundred and eleven days after transplanting MR219.

Days of sampling	Treatments			
	T1	T2	T3	T4
	mg kg^−1^			
Initial	115.53^c^ ± 3.32	141.81^b^ ± 2.42	188.34^a^ ± 1.17	122.19^c^ ± 2.53
10	59.31^d^ ± 1.34	153.28^c^ ± 1.69	175.13^b^ ± 5.09	198.50^a^ ± 3.12
20	36.19^d^ ± 0.70	68.42^c^ ± 4.30	84.00^b^ ± 2.75	172.63^a^ ± 1.64
30	37.30^d^ ± 1.71	68.66^c^ ± 1.47	84.83^b^ ± 1.20	174.08^a^ ± 0.73
40	53.49^c^ ± 3.49	58.59^c^ ± 1.28	152.5^a^ ± 1.34	140.50^b^ ± 6.01
50	48.52^c^ ± 2.82	48.83^c^ ± 2.30	125.42^a^ ± 2.83	91.71^b^ ± 3.01
60	45.59^c^ ± 1.58	72.04^b^ ± 2.531	95.28^a^ ± 7.17	90.65^a^ ± 4.69
70	35.49^d^ ± 2.83	53.51^c^ ± 4.51	84.39^b^ ± 4.05	95.97^a^ ± 0.39
80	37.59^c^ ± 0.99	61.91^b^ ± 2.63	63.25^b^ ± 2.46	120.19^a^ ± 6.96
90	47.45^c^ ± 1.18	52.75^c^ ± 1.84	84.67^a^ ± 3.50	66.19^b^ ± 2.14
100	34.68^a^ ± 1.69	38.93^a^ ± 2.48	–	–
111	33.31 ± 1.08	–	–	–

Different letters within a row indicates significant difference between means of four replicates ± standard error using Tukey’s test at *p* ≤ 0.05.

**Table 12:** Chicken litter biochar on available phosphorus at one hundred and eleven days after transplanting MR219.

Days of sampling	Treatments			
	T1	T2	T3	T4
	mg kg^−1^			
Initial	1.82^c^ ± 0.07	25.10^a^ ± 0.57	12.41^b^ ± 0.71	26.32^a^ ± 0.66
10	1.32^d^ ± 0.09	13.77^c^ ± 0.37	25.60^b^ ± 1.73	40.78^a^ ± 1.05
20	1.56^d^ ± 0.11	21.70^c^ ± 0.80	26.87^b^ ± 0.71	34.66^a^ ± 0.96
30	1.11^d^ ± 0.05	25.85^b^ ± 1.13	21.32^c^ ± 1.05	32.61^a^ ± 0.90
40	1.43^c^ ± 0.10	26.90^b^ ± 0.86	26.16^b^ ± 0.67	41.38^a^ ± 0.68
50	1.67^c^ ± 0.08	20.42^b^ ± 0.80	20.15^b^ ± 0.38	34.58^a^ ± 0.50
60	23.40^c^ ± 1.74	26.87^c^ ± 1.81	53.06^a^ ± 4.98	38.67^b^ ± 1.67
70	0.92^d^ ± 0.04	7.71^c^ ± 0.57	14.71^b^ ± 1.70	30.17^a^ ± 0.40
80	2.53^c^ ± 0.44	14.82^b^ ± 1.25	14.40^b^ ± 1.59	28.48^a^ ± 4.23
90	1.12^c^ ± 0.04	13.48^b^ ± 0.94	15.58^b^ ± 1.88	29.39^a^ ± 2.49
100	1.00^b^ ± 0.11	10.78^a^ ± 0.76	–	–
111	1.17 ± 0.07	–	–	–

Different letters within a row indicates significant difference between means of four replicates ± standard error using Tukey’s test at *p* ≤ 0.05.

Glaser et al.^60^ reported that chicken litter biochar increases soil nutrient retention and nutrient availability. Ch’ng et al.^44^ added that amending a tropical acid soil with chicken litter biochar increases availability of soil total P, available P, organic P, and inorganic fractions of P such as soluble-P, Al–P, Fe–P, redundant soluble-P, and Ca–P. This increase was associated with the reduction of soil exchangeable acidity, Fe, and Al. Furthermore, Brantley et al.^61^ reported that chicken litter biochar increased soil water-soluble P and soil total N because of the higher CEC of the chicken litter biochar which enables this organic amendment to electrostatically absorb or retain cations in soils^62^. Chicken litter biochars can also absorb or retains NO_3_^−^ and NH_4_^+^ to prevent these ions from being lost from soils^63–66^. A possible mechanism responsible for increasing N retention in soils which are amended with chicken litter biochar is the stimulation of microbial immobilisation of N and increased nitrates recycling because of higher availability of carbon. In lysimeter experiments, Lehmann et al.^67^ noticed that the ratio of uptake to leaching for all nutrients increased with biochar application to the soil. This observation is related to nutrient retention on the electrostatic adsorption complexes which are created by biochars. Steiner et al.^68^ attributed decreased leaching rates of applied mineral fertilizer N in soils which are amended with biochar to increased nutrient use efficiency.

### Influence of chicken litter biochar on nutrients uptake of MR219 rice

The increasing consumption of rice *vis a vis* the ever increasing human population is causing higher use of chemical fertilizers in rice cultivation in the tropics^69^. Consumption of these fertilizers is higher in tropical acid soils whose fertility is low. Apart from being acidic, these soils are low water holding capacity, cation exchange capacity (CEC), base saturation, and base cations^18,70^. In this present study, chicken litter biochar was used to improve nutrient availability and uptake. Nitrogen, P, K, S, Zn, Fe, Mn, and B fertilizers are among the commonly used fertilizers in lowland rice cultivation but, N, P, and K fertilizers account for approximately 92% of the major nutrients used in rice cultivation^69^. Among the three major elements, N is the highly used nutrient element because it plays an essential role in plant metabolism system^71^. This is because all vital processes in plants especially in rice production are associated with protein making N application in rice production indispensable^72^. Nitrogen contains essential amino acid which are involved in catalyzation of chemical responses and transportation of electrons that increases plant photosynthesis^73^.

**Figure 5:**
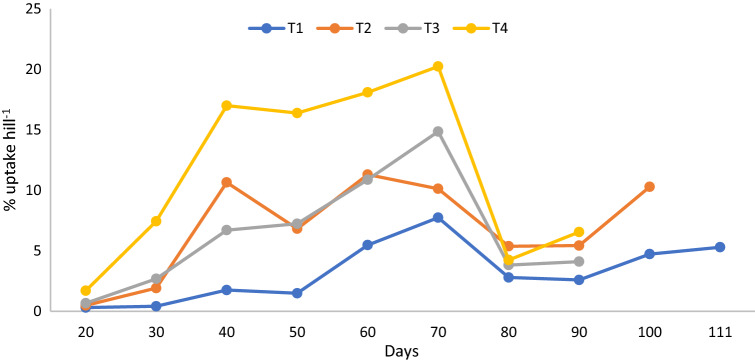
Chicken litter biochar on total nitrogen uptake of MR219 rice plants cultivated on a tropical acid soil.

Nitrogen in rice plant increases the physiological processes such as increasing dark-green color in plants, promotes leaves number, tillering, and other vegetative part’s growth and development^74^. Nitrogen also stimulates root growth of rice plant^75^. Nitrogen enables the uptake and utilization of other nutrients such as P and K^76^. In this present study, N and P uptake were determined at 10 days interval, however, on the 10th day after transplanting, there data were not recorded because the rice plants were small for harvest (Fig. 4). On day 20 after transplanting, the rice plants’ total N and P were analyzed, and the results demonstrated that the total N uptake of T4 was higher than those of T2, T3, and T4. Although urea was applied in the plots with T2 on day 15 (first stage of N application), it did not significantly increase N uptake of the rice plants on day 20 compared to T4 because of the influence of the chicken litter biochar on root growth and nutrient absorption. From days 30–40, the total N uptake of T4 was significantly higher than with T2 and N uptake plant of T2 was significantly higher than those with T1 and T3. This was because urea was applied to the plots with T2 on day 15 after transplanting whereas the T3 plots had lower N from the chicken litter biochar only to support the rice plants’ growth and development. Also, the results suggests that the urea in T2 and T4 significantly contributed to improvement in N uptake compared with those of T1 and T3 (Fig. 5).

From days 70 to 80, N uptake decreased regardless of treatment because this period marked the booting and panicle heading stages of the rice plants. Moreover, the rice plants have developed active root system at early and middle growth stages^77^. Additionally, studies have revealed a correlation between number of spikelets per panicle and absorbed N up to flowering stage^22,78^. This explains the decrease in nutrient uptake after 70 days of transplanting. Nitrogen accumulation from panicle initiation (PI) to flowering increases carbohydrate accumulation at heading and maturity stages and this translates into improved rice grain yield^79,80^. Generally, the N uptake for T3 was similar to that of the conventional method of producing rice (T2), suggesting the contribution of N uptake by the chicken litter biochar alone was similar to the N of the conventional method. Total P uptake of T3 and T4 were similar on day 20 but significantly higher than those of T1 and T2 (Table 13). From days 30–90, the total P uptake of T4 was significantly higher than those of T1, T2, and T3 (Table 13).

**Table 13:** Chicken litter biochar on plant uptake of total phosphorus at one hundred and eleven days of MR219 cultivation.

Days of sampling	Treatments			
	T1	T2	T3	T4
	mg kg^−1^			
20	118.27^a^ ± 3.53	680.51^b^ ± 27.75	2165.49^a^ ± 52.84	2271.72^a^ ± 58.39
30	361.55^d^ ± 17.87	1817.42^c^ ± 80.64	9724.62^b^ ± 381.05	17,966.82^a^ ± 320.68
40	777.89^d^ ± 81.31	4150.52^c^ ± 266.72	11,679.06^b^ ± 801.86	28,240.02^a^ ± 2255.29
50	1253.54^d^ ± 46.96	5473.68^c^ ± 42.15	18,991.85^b^ ± 575.79	36,273.77^a^ ± 1403.67
60	2730.33^d^ ± 111.34	12,331.21^c^ ± 450.04	28,756.57^b^ ± 917.05	48,345.41^a^ ± 1540.86
70	4590.87^d^ ± 134.60	16,552.82^c^ ± 581.05	28,091.79^b^ ± 1988.14	81,146.97^a^ ± 1903.48
80	3302.06^d^ ± 152.76	9709.01^c^ ± 215.87	15,184.51^b^ ± 1508.28	28,554.05^a^ ± 1631.96
90	4988.32^d^ ± 306.79	11,363.53^c^ ± 647.95	17,061.99^b^ ± 844.41	27,317.35^a^ ± 520.09
100	2304.48^b^ ± 153.46	10,346.25^a^ ± 173.22	–	–
111	2621.97 ± 44.77	–	–	–

Different letters within a row indicates significant difference between means of four replicates ± standard error using Tukey’s test at *p* ≤ 0.05.

The quality of the chicken litter biochar used in this study is related to its C:N ratio. C:N ratio of the chicken litter biochar leads to slower decomposition^81,82^. The slower degradation of the chicken litter biochar in the soil is of great importance in terms of absorption and desorption of plant nutrients from chemical fertilizers^83^. Chicken litter biochar are also resilient to microbial attack and this explains their longer resident period with organic amendments such as composts. This provides physical protection to soils, improves rice plant root development in addition to improving chemical fertilizer use efficiency^84^. Among the treatments evaluated in this presented study, T4 significantly increased N and P uptake. Reducing C:N ratio of chicken litter biochar by using urea might have increased the decomposition rate of the chicken litter biochar. Lower decomposition results low release of important nutrients for plants uptake. The co-application of the chicken litter biochar and chemical fertilizers significantly improved the plant total N and P uptake compared with the use of conventional method of cultivating rice.

### Growth variables of MR219 at maturity

Optimum nitrogen use in rice production increases photosynthetic processes, leaf area production, leaf area duration, and net assimilation rate^85^. The maximum leaf area (LA) and total leaf biomass of plants are a determinant of higher crop yield^86^. All plants including cereals, oilseeds, fibre, and sugar producing horticultural plants require a balanced amount of N for vigorous growth and development^87^. Efficient use of N ensures good harvest with better dry matter and grain yield^88,89^. In this present study, improving N use efficiency of rice plants through the use of chicken litter biochar significantly affected the rice plants’ growth variables. On day 20, the dry matter of T3 and T4 were similar but significantly higher than those of T1 and T2. From day 30 to day 70, the dry matter of T4 was significantly higher compared with those of T1, T2, and T3 but that of T3 was higher than with T2 and T1 (Table 14).

**Table 14:** Chicken litter biochar on plant dry matter yield at one hundred and eleven days of MR219 cultivation.

Days of sampling	Treatments			
	T1	T2	T3	T4
	g hill^−1^			
20	0.22^c^ ± 0.03	0.62^b^ ± 0.05	1.39^a^ ± 0.16	1.31^a^ ± 0.12
30	0.62^d^ ± 0.05	2.28^c^ ± 0.14	5.92^b^ ± 0.49	8.50^a^ ± 0.53
40	1.29^d^ ± 0.14	5.51^c^ ± 0.35	7.86^b^ ± 1.11	17.61^a^ ± 1.34
50	2.28^d^ ± 0.17	8.47^c^ ± 0.11	10.90^b^ ± 0.59	21.83^a^ ± 0.79
60	6.06^d^ ± 0.69	15.24^c^ ± 1.19	20.18^b^ ± 0.78	30.51^a^ ± 1.14
70	7.39^c^ ± 0.34	21.06^b^ ± 0.85	22.69^b^ ± 0.98	50.35^a^ ± 1.16
80	6.26^b^ ± 0.69	16.08^a^ ± 0.71	15.92^a^ ± 1.46	17.53^a^ ± 1.00
90	9.21^c^ ± 0.71	19.34^b^ ± 1.55	16.41^b^ ± 1.43	26.81^a^ ± 0.53
100	7.99^b^ ± 0.96	22.90^a^ ± 0.53	–	–
111	10.68 ± 0.72	–	–	–

Different letters within a row indicates significant difference between means of four replicates ± standard error using Tukey’s test at *p* ≤ 0.05.

**Figure 6:**
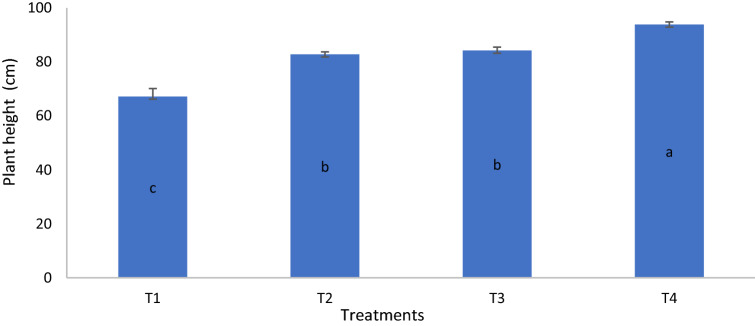
Chicken litter biochar on height of MR219 rice plants cultivated on a tropical acid soil.

**Figure 7:**
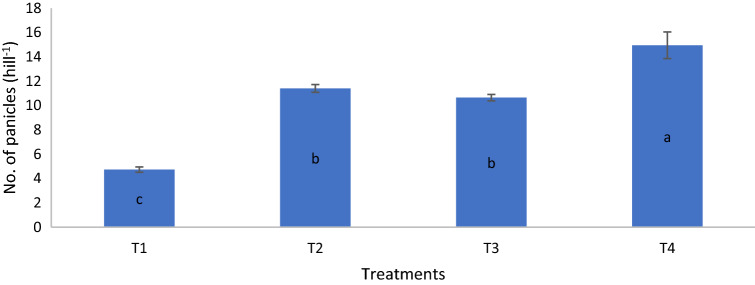
Chicken litter biochar on numbers of panicles of MR219 rice plants cultivated on a tropical acid soil.

**Figure 8:**
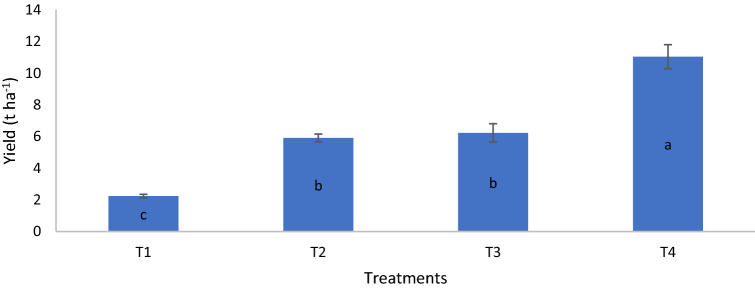
Chicken litter biochar on rice grain yield of MR219 rice plants.

**Figure 9:**
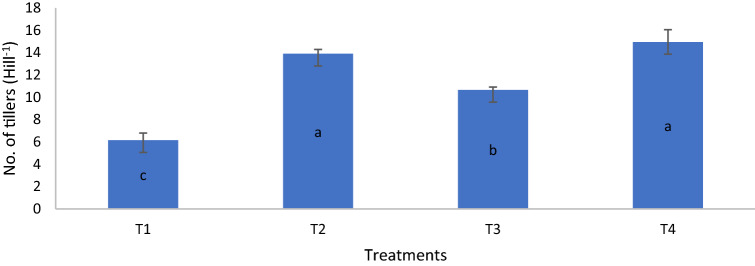
Chicken litter biochar on numbers of tillers of MR219 rice plants cultivated on a tropical acid soil.

On day 80, the dry matter yield of T4 was similar to those of T2 and T3 but significantly higher than that of T1. On day 90, the dry matter yield of T4 was significantly higher than those of T1, T2, and T3 whereas on day 100, the dry matter yield of T2 was significantly higher than that of T1 (Table 14). The growth variables and grain yield of MR219 at harvest revealed that height, number of panicles, and grain yield of the rice plants for T4 were significantly higher than those of T1, T2, and T3 whereas the height of T2 and T3 were similar but significantly higher than that of T1 (Figs. 6, 7, 8). The number of tillers of T4 and T2 were similar but significantly higher than those of T1 and T3 whereas the number of tillers of T3 was significantly higher than that of T1 (Fig. 9). The rice plant growth and development phases are as follows: (1) vegetative phase (from germination to panicle initiation stage, PI), (2) reproductive phase (from PI to flowering or heading stage), and (3) ripening phase (from flowering to maturity)^90,91^. These phases affect the three components of yield, namely: (a) number of panicles per unit land area, (b) average number and size of spikelets per panicle, and (c) average weight of individual grains^92–94^.

The early maturity of the rice plants observed in T4 and T3 was because the chicken litter biochar enhanced good tillering, internode elongation, and PI growth, approximately the same time^95,96^. Soil fertility at each of these phases affects rice plants’ growth variables and yield component^96–98^. There is a close correlation between number of spikelets per panicle and N absorption up to flowering^22,78^. Nitrogen accumulation from PI to flowering increases carbohydrate accumulation both at heading and at maturity, thereby increasing grain yield especially in T4^79,80^. The increase in N use efficiency of the rice plants especially for T4, increased the rice grain yield because of the rice plants demand for higher N uptake at early tillering stage to increase panicle formation and at reproductive and ripening phases to maximize spikelets per panicle and percentage filled per spikelets^1,7^.

A study by Palanivell et al.^12^ on a tropical acid soil revealed significant increase in the yield of MR219 (Malaysia hybrid rice) to approximately 10 t ha^−1^ using chicken litter biochar. Following application of chicken litter biochar, Maru et al.^45^ reported that urea use in cultivating MR219 can be reduced by 25% at same the time increase rice yield from 4 t ha^−1^ (existing yield) to 8.4 t ha^−1^ (potential yield). In general, hybrids of any crop are more responsive to high N levels. The physiological basis for higher response to N by hybrid rice is its capacity to increase photosynthetic activity by increasing leaf area (LA), thereby resulting in more accumulation of dry matter^99^.

### Crop recovery and agronomic efficiency of spilt urea application

**Figure 10:**
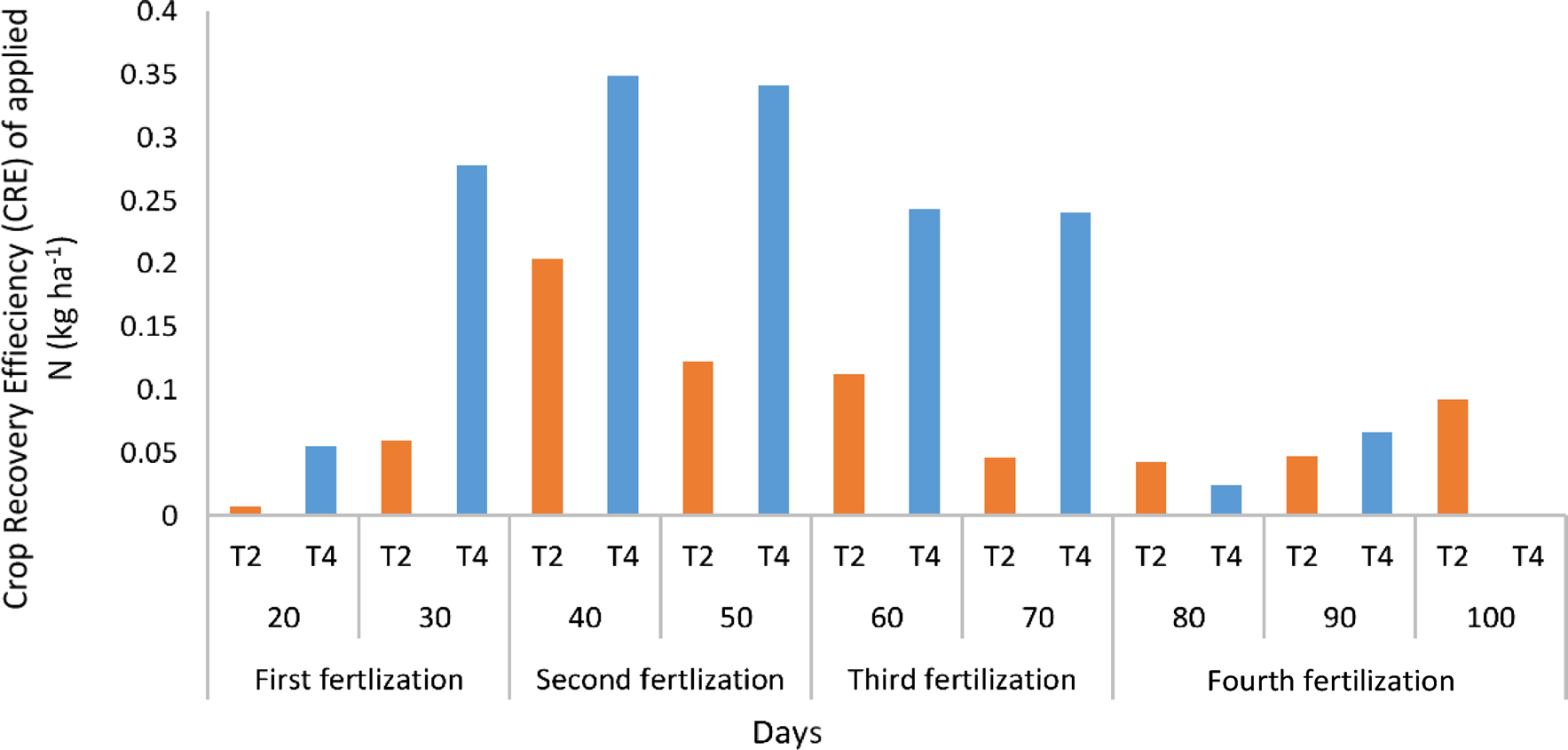
Chicken litter biochar on crop recovery efficiency (RE) of applied nitrogen.

**Figure 11:**
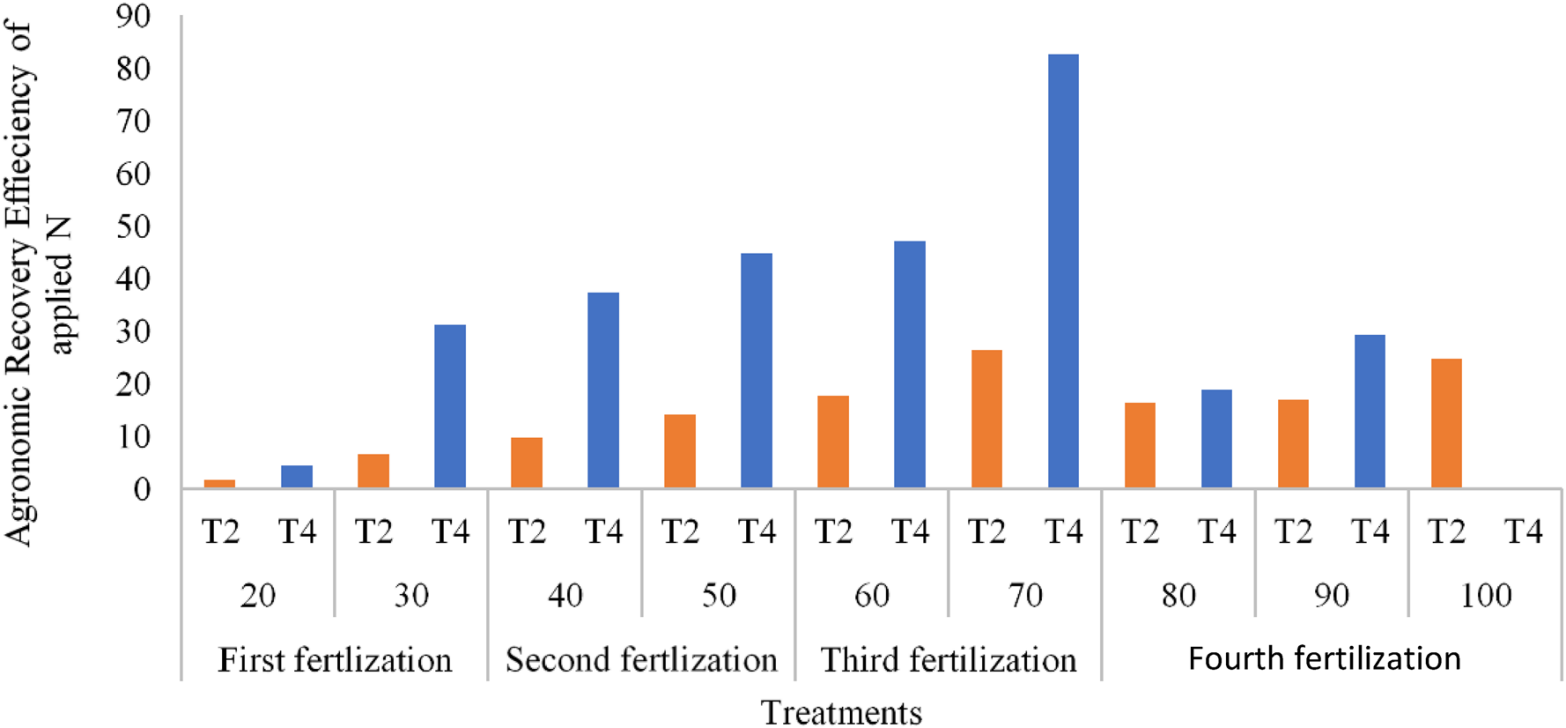
Chicken litter biochar on agronomic recovery efficiency (ARE) of applied nitrogen.

Nitrogen being a macro nutrient can be highly available in soils at higher pH. However, this availability does not guarantee higher N use efficiency because soil pH exceeding for example, seven may increase ammonia volatilization if the N is not timely absorbed by plants^100^. The use of chicken litter biochar in this present study significantly increased crop recovery efficiency (RE_N_) and agronomic recovery efficiency (ARE) of applied N. The results of the study showed that both RE_N_ and ARE of T4 were significantly higher than that of T2 on days 20, 30, 40, 50, 60, 70, 80, and 90 (Tables 15, 16, 17, 18, 19, 20, 21, 22, 23). The RE_N_ and ARE of T4 increased with increasing days than with T2. However, after 40 days, RE_N_ decrease with a significant reduction on day 70 after transplanting. A similar trend was observed for ARE although the decrease for T4 occurred after 70 days of transplanting. The highest ARE occured on day 70 after which there was a significant decrease of ARE from days 80–90 (Figs. 10, 11) because the rice plant had reached panicle heading stage (70 days after transplanting).

**Table 15:** Chicken litter biochar application and split application of urea on crop recovery and agronomic efficiency on day twenty after transplanting.

Treatments	Total N applied	Plant total N uptake	Plant dry matter yield	Crop recovery efficiency of applied N	Agronomic efficiency of applied N
Days	Twenty days after transplanting				
	kg ha^−1^				
T1	0	0.765802	55	–	–
T2	63.25	1.211077	155	0.00704	1.581028
T3	0	1.671724	347.5	–	–
T4	63.25	4.247044	327.5	0.055039	4.3083

**Table 16:** Chicken litter biochar application and split application of urea on crop recovery and agronomic efficiency on day thirty after transplanting.

Treatments	Total N applied	Plant total N uptake	Plant dry matter yield	Crop recovery efficiency of applied N	Agronomic efficiency of applied N
Days	Thirty days after transplanting				
	kg ha^−1^				
T1	0	1.030698	155	–	–
T2	63.25	4.788355	570	0.05941	6.561265
T3	0	6.713572	1480	–	–
T4	63.25	18.59667	2125	0.277723	31.14625

**Table 17:** Chicken litter biochar application and split application of urea on crop recovery and agronomic efficiency on day forty after transplanting.

Treatments	Total N applied	Plant total N uptake	Plant dry matter yield	Crop recovery efficiency of applied N	Agronomic efficiency of applied N
Days	Forty days after transplanting				
	kg ha^−1^				
T1	0	4.39161	322.5	–	–
T2	109.25	26.63043	1377.5	0.203559	9.656751
T3	0	16.77026	1965	–	–
T4	109.25	42.49899	4402.5	0.348809	37.34554

**Table 18:** Chicken litter biochar application and split application of urea on crop recovery and agronomic efficiency on day fifty after transplanting.

Treatments	Total N applied	Plant total N uptake	Plant dry matter yield	Crop recovery efficiency of applied N	Agronomic efficiency of applied N
Days	Fifty days after transplanting				
	kg ha^−1^				
T1	0	3.724471	570	–	–
T2	109.25	17.05323	2117.5	0.122002	14.16476
T3	0	18.09221	2725	–	–
T4	109.25	40.96535	5457.5	0.340878	44.73684

**Table 19:** Chicken litter biochar application and split application of urea on crop recovery and agronomic efficiency on day sixty after transplanting.

Treatments	Total N applied	Plant total N uptake	Plant dry matter yield	Crop recovery efficiency of applied N	Agronomic efficiency of applied N
Days	Sixty days after transplanting				
		kg ha^−1^			
T1	0	13.68222	1515	–	–
T2	129.95	28.25204	3810	0.112119	17.66064
T3	0	27.19837	5045	–	–
T4	129.95	45.24048	7627.5	0.242849	47.03732

**Table 20:** Chicken litter biochar application and split application of urea on crop recovery and agronomic efficiency on day seventy after transplanting.

Treatments	Total N applied	Plant total N uptake	Plant dry matter yield	Crop recovery efficiency of applied N	Agronomic efficiency of applied N
Days	Seventy days after transplanting				
	kg ha^−1^				
T1	0	19.35482	1847.5	–	–
T2	129.95	25.32194	5265	0.045919	26.29858
T3	0	37.14157	5672.5	–	–
T4	129.95	50.5877	12587.5	0.240345	82.64717

**Table 21:** Chicken litter biochar application and split application of urea on Crop Recovery and Agronomic Efficiency on day eighty after transplanting.

Treatments	Total N applied	Plant total N uptake	Plant dry matter yield	Crop recovery efficiency of applied N	Agronomic efficiency of applied N
Days	Eighty days after transplanting				
	kg ha^−1^				
T1	0	6.983351	1565	–	–
T2	150.65	13.43559	4020	0.042829	16.29605
T3	0	9.547838	3980	–	–
T4	150.65	10.59725	4382.5	0.023989	18.70229

**Table 22:** Chicken litter biochar application and split application of urea on crop recovery and agronomic efficiency on day ninety after transplanting.

Treatments	Total N applied	Plant total N uptake	Plant dry matter yield	Crop recovery efficiency of applied N	Agronomic efficiency of applied N
Days	Ninety days after transplanting				
	kg ha^−1^				
T1	0	6.470212	2302.5	–	–
T2	150.65	13.57698	4835	0.047174	16.81049
T3	0	10.26386	4102.5	–	–
T4	150.65	16.3783	6702.5	0.065769	29.20677

**Table 23:** Chicken litter biochar application and split application of urea on crop recovery and agronomic efficiency on day one hundred after transplanting.

Treatments	Total N applied	Plant total N uptake	Plant dry matter yield	Crop recovery efficiency of applied N	Agronomic efficiency of applied N
Days	Hundred days after transplanting				
	kg ha^−1^				
T1	0	11.81507	1997.5	–	–
T2	150.65	25.71242	5725	0.092249	24.74278
T3	0	–	–	–	–
T4	150.65	–	–	–	–

Several factors in rice production influence N use efficiency and among these factors are age of rice plant, rice plant type, soil type, and environmental parameters^101^. Among these factors, soil and climatic factors are most considered in N use efficiency in rice plant growth and development whereas age of rice plant and its genetic characteristics are not considered in any agricultural discipline except by rice plant breeders^102^. The use of urea at initial the growth phase of the rice plants did not significantly increase N-use efficiency because the rice plants had not developed enough rooting system for N absorption^103^. However, in the course of the rice plants growth and development, N use and crop recovery efficiency increased until the end of booting stage (70 days after transplanting). Baligar and Fageria^104^ uncovered that, soil pH, soil texture, structure, soil compaction, organic matter, moisture, and presence of other nutrients affect N-use efficiency of rice plants because the hydrogen ions or hydroxyl ions in the soil solution interfere with N utilization by the rice plants. The lower the hydrogen ions in the soil solution, the higher the N use efficiency of rice plants (pH 6.5–7)^105^. This confirms the higher RE_N_ and ARE of T4 relative to T2.

Conditioning soils with organic amendments ultimately improves N efficacy by supporting utilization of N, thereby improving rice plant root growth and rice nutrient used efficiency. Plant breeding and genetic engineering have resulted in developing many high yielding rice varieties which respond well to N. This response improves N use^106^. The decrease in RE_N_ and ARE after 70 days of transplanting might be due to the higher demand of other nutrient elements by the rice plants for grain filling (Figs. 10, 11). This is because the chemical reaction among the elements which enable loss or gain of electron(s) changes the shape of some nutrient elements thereby reducing N use efficiency in rice plants at maturity stage. Also, the slower root activity of rice plants at maturity might be a contributing factor to the decrease in N use efficiency of the rice plants at maturity because the rice plants’ active root system at early and middle growth stages compared with the maturity stage^77^.

## Conclusion

The use of chicken litter biochar increases soil pH, total carbon, total P, available P, total N, and exchangeable N. Moreover, this organic amendment decreases soil total acidity and exchangeable Al^3+^. For T4 in particular, the improved soil chemical properties following the application of chicken litter biochar caused significant improvement in Crop Recovery Efficiency and Agronomic Recovery Efficiency of N relative to the existing method without chicken litter biochar (T2). This resulted in significant increase in the yield (11 t ha^−1^) of MR219 (Malaysia hybrid rice) for T4 compared with the existing yield of 5.9 t ha^-1^ (T2). Moreover, the existing N application of rates of 41.67%, 30.03%, 14.15%, and 14.15% on days 15, 35, 55, and 75, respectively should be replaced with N application rates of 26.67%, 30.03%, 30.15%, and 14.15% on days 10, 30, 50, and 70, respectively. Chicken litter biochar can improve the chemical properties of tropical acid soils and yield rice (MR219) cultivation.
